# Genetic Control of Lithium Sensitivity and Regulation of Inositol Biosynthetic Genes

**DOI:** 10.1371/journal.pone.0011151

**Published:** 2010-06-17

**Authors:** Jason King, Melanie Keim, Regina Teo, Karin E. Weening, Mridu Kapur, Karina McQuillan, Jonathan Ryves, Ben Rogers, Emma Dalton, Robin S. B. Williams, Adrian J. Harwood

**Affiliations:** 1 School of Biosciences, Cardiff University, Cardiff, United Kingdom; 2 School of Biological Sciences, Royal Holloway, University of London, Egham, Surrey, United Kingdom; CNRS UMR6543, Université de Nice, Sophia Antipolis, France

## Abstract

Lithium (Li^+^) is a common treatment for bipolar mood disorder, a major psychiatric illness with a lifetime prevalence of more than 1%. Risk of bipolar disorder is heavily influenced by genetic predisposition, but is a complex genetic trait and, to date, genetic studies have provided little insight into its molecular origins. An alternative approach is to investigate the genetics of Li^+^ sensitivity. Using the social amoeba *Dictyostelium*, we previously identified prolyl oligopeptidase (PO) as a modulator of Li^+^ sensitivity. In a link to the clinic, PO enzyme activity is altered in bipolar disorder patients. Further studies demonstrated that PO is a negative regulator of inositol(1,4,5)trisphosphate (IP_3_) synthesis, a Li^+^ sensitive intracellular signal. However, it was unclear how PO could influence either Li^+^ sensitivity or risk of bipolar disorder. Here we show that in both *Dictyostelium* and cultured human cells PO acts via Multiple Inositol Polyphosphate Phosphatase (Mipp1) to control gene expression. This reveals a novel, gene regulatory network that modulates inositol metabolism and Li^+^ sensitivity. Among its targets is the inositol monophosphatase gene IMPA2, which has also been associated with risk of bipolar disorder in some family studies, and our observations offer a cellular signalling pathway in which PO activity and IMPA2 gene expression converge.

## Introduction

Bipolar mood disorder is a common psychiatric disorder, which is treated using mood stabilizer drugs, such as lithium (Li^+^). There is a strong genetic component to risk of developing bipolar disorder, however this is a complex genetic trait and no consensus candidate susceptibility genes have yet emerged from those identified in individual family studies [1]. Genetics is not the only factor affecting risk, and twin studies demonstrate the presence of environmental inputs [2], [3]. Environmental risk factors include lifetime events and psychosocial stressors, physical illnesses and hormonal imbalances [4]. Onset of bipolar disorder is most frequent during late adolescence and early adulthood suggesting contributions from developmental and physiological mechanisms. As in the case of genetics, the biological mechanisms underlying environmental risk are also unclear.

An alternative approach is to investigate the therapeutic mechanisms of the mood stabilizer drugs used to treatment bipolar disorder. Li^+^ is the most widely used mood stabilizer, taken by 50% of bipolar patients. It is an effective prophylactic agent and good anti-manic, but a weaker anti-depressant treatment [5]. Inositol monophosphatase (IMPase) and Inositol 1-polyphosphatase (IPP) are direct biochemical targets of Li^+^ [6], [7], and Li^+^ treatment leads to reduced inositol synthesis and suppression of inositol phosphate (IP) signalling. Consequently, it has been proposed that the neurological processes underlying bipolar mood disorder are susceptible to aberrant IP signalling [8]. Consistent with this hypothesis, the structurally unrelated mood stabilizers valproic acid (VPA) and carbamazepine also deplete inositol [9], [10], indicating a wider association between mood stabilizers and IP signalling.

To understand why Li^+^ is an effective therapy, we undertook a genetic screen for Li^+^ resistance in the social amoeba *Dictyostelium*. This identified the serine protease prolyl oligopeptidase (PO) as a modulator of Li^+^ sensitivity [11]. Interestingly, reduced PO activity has been associated with bipolar disorder [12], [13], [14]. We found that ablation of the PO gene or inhibition of PO enzyme activity is accompanied by an increase in inositol(1,4,5)trisphosphate (IP_3_) production. This inhibitory interaction is conserved in mammalian cells as reduced PO activity increases IP_3_ in astroglioma and COS-7 kidney cell lines [10], [15]. These effects are manifest at the cellular level where PO inhibition both reverses the effects of Li^+^, VPA and carbamazepine on neuronal growth cone morphology and stimulates removal of protein aggregates via macro-autophagy [9], [10], [16], [17].

The mechanism by which PO controls Li^+^ sensitivity has not been established. Initial *Dictyostelium* studies showed that PO null mutants had unaltered phospholipase C activity but increased activity of two inositol phosphate phosphatases. One phosphatase activity corresponds to multiple inositol polyphosphate phosphatase (Mipp1), which dephosphorylates IP_6_ to IP_3_. The other is an IP_3_ 5-phosphatase activity that dephosphorylates IP_3_ further to I(1,4)P_2_ [11]. Here, we report that Mipp1 is required to mediate the effects of PO on Li^+^ sensitivity. Furthermore, we show that these effects do not arise through direct elevation of IP_3_ production as previously assumed, but are due to changes in the expression of genes involved in inositol synthesis and metabolism, including inositol synthase (*ino1*), IMPase (*impA1*) and the *Dictyostelium* IP_3_ 5-phosphatase (Dd5P3). Finally, we show that this PO mediated regulation of gene expression is conserved in human cells where among its targets is IMPA2, a gene associated with risk in some bipolar disorder patient family studies. Our observations suggest a signalling pathway that could connect altered PO mediated signalling to aberrant IMPA2 gene regulation.

## Results

### *Dictyostelium* PO, *dpoA*, reduces the effect of Li^+^ on chemotaxis

**Figure 1:**
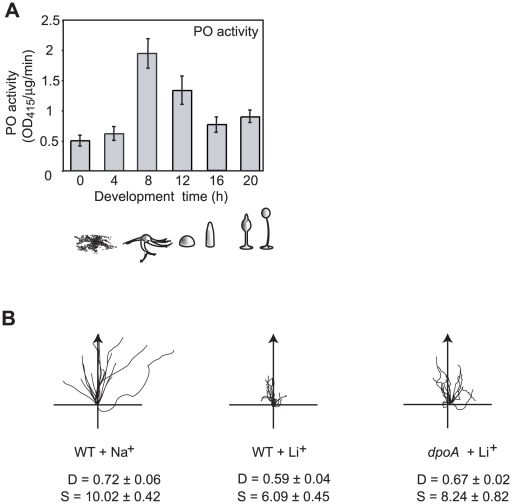
Loss of *dpoA* reduces Li^+^ sensitivity. **A**, PO activity peaks at 8 hours of *Dictyostelium* development, and then declines to levels only slightly higher than in growing cells (0 hours of development). Error Bars: mean ± standard error of 3 independent experiments. Peak activity corresponds to streaming and mound developmental stages. **B**, Cell tracks of wild type and mutant cells in 7 mM LiCl (or control of 7 mM NaCl) during chemotaxis along a 1 nM/µm cAMP gradient (direction of arrow). D =  Directionality, S =  cell speed (µm/min). *dpoA* null mutants have a reduced Li^+^ sensitivity, showing higher Directionality and speed than wild type in Li^+^.

Starved *Dictyostelium* cells aggregate by cAMP-mediated chemotaxis to form a multicellular mass of 100,000 cells, which develops further into a fruiting body of spore and stalk cells [18]. We found that PO activity is regulated throughout *Dictyostelium* development, peaking during late aggregation and early multicellular stages (Figure 1A), indicative of a regulatory function.

Li^+^ treatment of wild type cells has a specific effect on chemotaxis, causing reduced cell speed and Directionality (a measure of the linearity of the cell path) [19]. We found that chemotaxis of *dpoA* mutant cells was less sensitive to Li^+^, with cells exhibiting greater speed and Directionality in the presence of Li^+^ than wild type cells (Figure 1B; Table 1). We also noted that *dpoA* mutant cells exhibited increased speed of movement in control conditions, treatment with NaCl, suggesting that DpoA suppresses this aspect of the chemotaxis response.

**Table 1:** Chemotactic analysis of lithium sensitivity.

				Relative lithium sensitivity	
Strain		Directionality (D)	Speed (s) (µm/min)	Directionality	Speed
Wild type	Na	0.72±0.06	10.02±0.42		
	Li	0.59±0.04††	6.09±0.45†††		
*dpoA*	Na	0.68±0.02	11.34±0.57§		
	Li	0.67±0.02***	8.24±0.82*	++	++
*mipp1*	Na	0.69±0.02	9.65±0.19		
	Li	0.50±0.06†††	4.69±0.58†††	- -	- -
*mipp1:dpoA*	Na	0.73±0.03	9.62±0.27		
	Li	0.51±0.04†††	5.46±0.22†††,$	- -	-
*mipp1* ^oe^	Na	0.68±0.03	9.02±0.79		
	Li	0.46±0.04†††	2.96±0.12†††	- - -	- - -
*ImpA1^oe^*	Na	0.70±0.06	9.80±0.60		
	Li	0.66±0.05*	7.85±0.43**	++	++
*ImpA1^oe^:Ino1^oe^*	Na	0.51±0.02	18.89±0.84		
	Li	0.41±0.03	8.48±0.46*	- - -	++
*ipkA1* ^oe^	Na	0.77±0.05	10.03±0.80		
	Li	0.68±0.03***	10.07±0.65***	++	+++
*ipkB* ^oe^	Na	0.69±0.01	10.03±0.22		
	Li	0.71±0.03***	9.08±0.95***	+++	++
*mipp1:ipkA1* ^oe^	Na	0.65±0.02	8.09±0.59		
	Li	0.36±0.04	4.64±0.60	- - - -	-
*mipp1:ipkB* ^oe^	Na	0.62±0.11	8.74±1.79		
	Li	0.40±0.01	5.43±0.54	- - - -	-

Cells were treated with 7mM LiCl or NaCl (control treatment) for one hour prior to analysis. Values shown are the mean ± standard error of data from at least three independent experiments. Directionality is defined as the total distance moved divided by the net distance moved. Statistical analysis: two-tailed t-test.

†††p<0.001,

††p<0.02, compared to NaCl control treatment.

***p<0.005,

**p<0.05,

*p<0.1, compared to LiCl-treated wild-type cells.

§p<0.001, compared to NaCl treated wild type cells. $ p<0.05, compared to LiCl treated *dpoA* null cells.

Relative lithium sensitivity

This is based on the difference between the numerical values for lithium treatment of wild type and mutant cells, as calculated by (value of mutant in Lithium)/(value for WT in Lithium). For Directionality: (- - -) 60–69% of wild type; (- - -) 70–79% of wild type; (- -) 80–89% of wild type; (++) 110–119% of wild type; (+++) 120–129% of wild type. For speed: (- - -) 25–49% of wild type; (- -) 50–74% of wild type; (-) 75–99% of wild type; (++) 125–149% of wild type; (+++) 150–174% of wild type.

### *Dictyostelium* PO acts via Multiple Inositol Polyphosphate Phosphatase (Mipp1)

**Figure 2:**
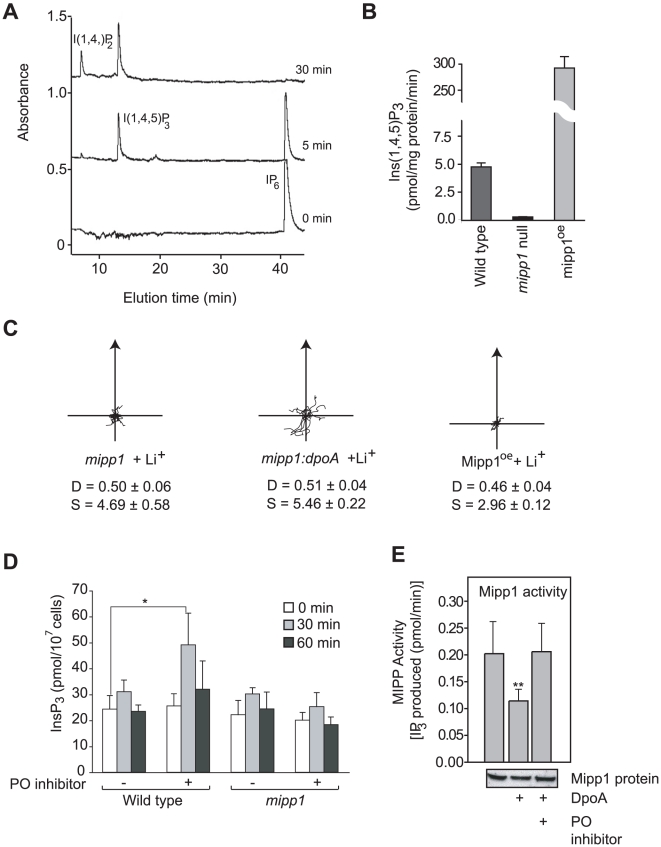
Mipp1 mediates the effects of PO inhibition. **A**, Mipp1 enzyme prepared from Mipp1 over-expressing cells were incubated with 10 nmoles InsP_6_ for the indicated times and analyzed by HPLC-MDD. I(1,4)P_2_ was generated by contaminating IP_3_ 5-phosphatase activity, and demonstrates that the product of Mipp1 is I(1,4,5)P_3_. **B**, Mipp1 activity measured as the rate of IP_3_ formation in extracts from wild type, *mipp1* null mutant and Mipp1overexpressingcells. **C**, Cell tracks of wild type and *mipp1* mutant cells in 7 mM LiCl (or control of 7 mM NaCl) during chemotaxis. *mipp*, *mipp:dpoA* and Mipp1 over-expressing mutants (Mipp1^oe^) are Li^+^ hypersensitive compared to wild type. D  =  Directionality, S =  cell speed (µm/min). **D**, Vegetative wild type and *mipp1* null cells were treated with either 1.2 mM Z-pro-L-prolinal or an equal volume of DMSO carrier as a control. Samples were then removed at the times indicated and IP_3_ concentration measured. Values plotted as mean ± standard error of 4 independent experiments. * P<0.05, paired T-test. **E**, Mipp1 protein extracts were incubated with recombinant DpoA with or without the PO inhibitor Z-pro-L-prolinal. Mipp1 activity is measured by the production of IP_3_ from IP_6_. Values plotted as mean ± standard error of 3 independent experiments. ** P<0.01, paired T-test. Samples of Mipp1 protein are shown on Western (underneath).

Previous results indicated that loss of *dpoA* is associated with increased activity of multiple inositol polyphosphate phosphatase (MIPP), an enzyme that dephosphorylates IP_6_, IP_5_ and certain IP_4_ species to form IP_3_ [11]. The *Dictyostelium* genome contains two homologues of the mammalian MIPP, however only one gene, denoted *mipp1*, is expressed in growing cells and during development (data not shown). We generated null mutant cell strains, which lack the *mipp1* gene, and strains that over-expressed *Dictyostelium* Mipp1. These latter strains were a useful source of Mipp1 enzyme, which was used to show that Mipp1 rapidly converts IP_6_ to IP_3_ (Figure 2A). *mipp1* null mutants completely lack Mipp activity and Mipp1 over-expressors greatly enhanced activity (Figure 2B). However, total mass measurement of all inositol phosphates (IPs) showed that loss of *mipp1* had no significant effect on the overall concentrations of IP_4_, IP_5_ and IP_6_ (data not shown) suggesting that Mipp1 regulates only a small proportion of the cellular IPs. Such robustness in the cellular content of the higher order IPs has been observed previously and means that small changes of the IP species involved in signalling may be masked behind larger non-dynamic IP populations [20].

Chemotaxis of *mipp1* null mutants was hypersensitive to Li^+^ (Figure 2C), the opposite phenotype of *dpoA* mutant cells. Double *dpoA:mipp1* null mutant cells possess a similar Li^+^ hypersensitive phenotype to *mipp1* null cells (Figure 2C), indicating that Mipp1 activity is required for PO mediated signalling. Consistent with this we found that elevation of IP_3_ following PO inhibition was also dependent on Mipp1. The IP_3_ concentration of wild type cells doubled within 30 minutes of addition of the PO inhibitor Z-pro-L-prolinal (Figure 2D). This increase was transient and IP_3_ returned to basal levels after 60 minutes. PO inhibitors were unable to elevate IP_3_ in *mipp1* null mutants (Figure 2D).

To investigate the interaction between PO and Mipp1 at the biochemical level, we pre-incubated Mipp1 extracts with bacterially produced recombinant DpoA protein, and then monitored Mipp1 activity. Incubation with DpoA enzyme caused an approximate two-fold decrease in Mipp1 activity, an effect reversed by treatment with PO inhibitor (Figure 2E). However, there was no change in the abundance or size of the Mipp1 protein following PO treatment (Figure 2E), arguing against direct proteolysis. Mipp1 is extracted as a membranous particulate fraction and the most likely explanation is that PO acts on a yet unidentified, co-purified intermediate protein or peptide. These results however do indicate a close association between PO activity and Mipp1 regulation.

### Over-expression of Mipp1 causes Li^+^ hypersensitivity

A simple hypothesis is that decreased PO activity controls Li^+^ sensitivity through Mipp1 mediated changes in IP_3_ abundance, but a number of observations show this is not the case. First, Mipp1 over-expression would be expected to increase the IP_3_ concentration and reduce Li^+^ sensitivity, however Mipp1 over-expressing cells are strongly Li^+^ hypersensitive (Figure 2C). Second, in the absence of PO inhibition, we found that neither loss nor over-expression of Mipp1 altered the cellular concentration of IP_3_, despite both causing Li^+^ hypersensitivity (data not shown). This lack of correlation argues against generation of IP_3_ as a direct mechanism for Li^+^ resistance. The same lack of reciprocity also argues against a mechanism involving depletion of the Mipp1 substrate IP_6_ or its alternative substrate 2,3-bisphosphoglycerate (2,3-BPG) [21]. I(1,3,4,5,6)P_5_ and I(1,4,5,6)P_4_/I(1,3,4,5)P_4_ are intermediates in the dephosphorylation of IP_6_ to I(1,4,5)P_3_ by Mipp1 and both have signalling properties within the cell. Mipp1 could therefore play a more complex role by regulating the abundance of these molecules through controlling the balance of their synthesis and degradation.

### Over-expression of the IP_3_ kinases IpkA1 and IpkB confers Li^+^ resistance

**Figure 3:**
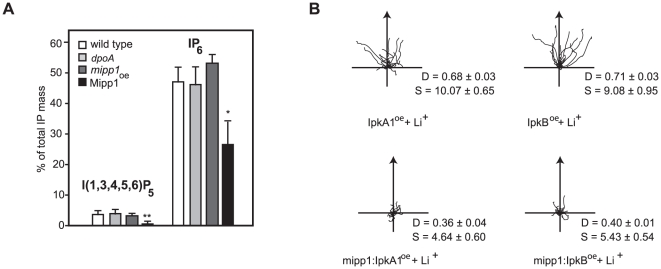
Elevated expression of Mipp1 and IP_3_ kinases alter lithium sensitivity. **A**, Comparison of the I(1,3,4,5,6)P_5_ and IP_6_ concentration of wild type, *dpoA* null; *mipp1* null and Mipp1 over-expressing cells. Values plotted (means ± standard error of 3 independent experiments) as percentage of the total IP content relative to wild type (*P<0.05, ** P<0.01 T-test). **B**, Cell tracks of wild type and *mipp1* null mutant cells over-expressing IpkA1 and IpkB during chemotaxis in 7 mM LiCl. D =  Directionality, S =  cell speed (µm/min). Over-expression of IpkA1 and IpkB in wild type cells confers Li^+^ resistance, whereas Li^+^ hypersensitivity is maintained after over-expression in a *mipp1* null mutant.

In all but one case, it was not possible to observe direct changes in the abundance of IPs in our mutants, however the exception was seen in Mipp1 over-expressing cells, which exhibited a large decrease in the cellular I(1,3,4,5,6)P_5_ concentration, in addition to decreased IP_6_ (Figure 3A). As this correlates with a strong Li^+^ hypersensitive phenotype, it suggested a positive role of I(1,3,4,5,6)P_5_ signalling in establishing Li^+^ sensitivity. To pursue this possibility, we over-expressed two structurally distinct IP_3_ kinases (IpkA1 and IpkB) each capable of generating I(1,3,4,5,6)P_5_ [22], [23], [24]. In the reverse of that seen for Mipp1 over-expression, chemotaxis of cells over expressing either IpkA1 or IpkB was resistant to Li^+^ (Figure 3B). Interestingly, we found that the Li^+^ resistant phenotype was lost when the IpkA1 or IpkB were over-expressed in a *mipp1* null mutant background (Figure 3B), arguing that Mipp1 activity may be the key regulator of this signal pathway.

### DpoA acts via Mipp1-mediated gene regulation

**Figure 4:**
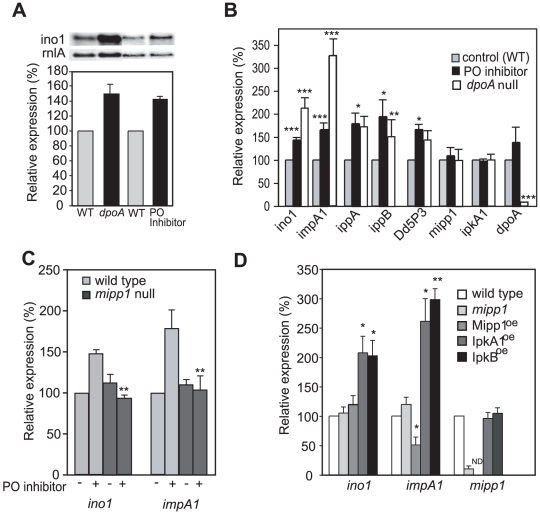
PO regulates gene expression. **A**, Expression of *ino1* in wild type, *dpoA* null cells and wild type cells following overnight treatment with 1.2 mM Z-pro-L-prolinal (PO inhibitor), measured by Northern analysis. *rnlA* expression was used as a loading control. The graph shows the expression of *ino1* quantified on a phosphoimager (Biorad) and normalised to *rnlA* relative to wild type or DMSO carrier treated controls (mean ± standard error of 3 independent experiments). **B**, The expression of genes involved in inositol metabolism (Table S1). Gene expression in wild type cells following overnight treatment with Z-pro-L-prolinal or the equivalent amount of DMSO carrier, and *dpoA* null mutant cells was measured by qRT-PCR, and normalised to *rnlA* expression levels. Values plotted are relative to the carrier control cells (mean ± standard error of 3 independent experiments). * P<0.05, ** P<0.01, *** P<0.005, T-Test. **C**, *mipp1* is required for the regulation of gene expression by PO. Expression of *ino1* and *impA1* in wild type and *mipp1* null cells following overnight treatment with Z-pro-L-prolinal or the equivalent amount of DMSO carrier, was measured by qRT-PCR, and normalised to *rnlA* expression levels. Values plotted are relative to the carrier control wild type cells (mean ± standard error of at least 3 independent experiments). There is a significant difference in gene expression between wild type and *mipp1* null mutant following PO inhibition (** P<0.01, T-Test). **D**, Expression levels of *ino1*, *impA1* and *mipp1* in wild type, *mipp1* null mutant and cells over-expressing Mipp1, IpkA1 or IpkB. Expression was calculated relative to either wild type, or wild type cells transformed with the empty expression vector as appropriate. All samples were normalised to *rnlA* as a loading control and the values plotted are the mean ± standard error of 3 independent experiments (*P<0.05, ** P<0.01 T-test). ND  =  not determined.

As the yeast homologue of *ipkA1*, Ipk2/Arg82, controls expression of the inositol synthase gene (*ino1*) [25], we investigated potential changes in gene expression mediated by PO and Mipp1. Expression of the *Dictyostelium ino1* gene [9], [26] was elevated both in *dpoA* null cells and cells treated with PO inhibitor (Figure 4A,B). Furthermore both conditions gave a 60-100% increase in expression of the genes encoding IMPase (*impA1*), IPP (*ippA* and *ippB*) and Dd5P3, a phosphatase that converts I(1,4,5)P_3_ to I(1,4)P_2_ (see Table S1). Dd5P3 is homologous to the synaptojanin-like IP_3_ 5-phosphatase conserved from yeast to humans [27] and its elevated expression is consistent with the elevated IP_3_ 5-phosphatase activity previously observed in *dpoA* null cells [11]. No change was seen for *mipp1*, *dpoA* or *ipkA1* genes (Figure 4B). Changes in gene expression were dependent on Mipp1, as *mipp1* null mutant cells treated with the PO inhibitor exhibited no change in the expression of *ino1* or *impA1* (Figure 4C). Furthermore, in the absence of PO inhibitor, Mipp1 over-expression caused a 50% decrease in expression of *impA1*, although *ino1* was unaffected (Figure 4C). Finally, IpkA1 and IpkB over-expression elevated *ino1* and *impA1* gene expression in the absence of PO inhibition (Figure 4D), again supporting a signalling role of the higher order IPs.

**Figure 5:**
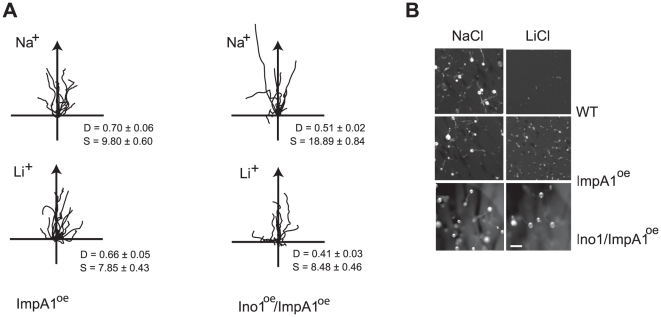
Increased *ino1* and *impA1* gene expression confers Li^+^ resistance. **A**, Cell tracks of wild type cells over-expressing ImpA1 alone or both ImpA1and ino1 undergoing chemotaxis in 7 mM NaCl or LiCl. D =  Directionality, S =  cell speed (µm/min). **B**, Cells of wild type cells over-expressing ImpA1 alone or both ImpA1and ino1were developed in the presence of 10 mM LiCl. Images are taken after 24 hours, bar  = 5 mm.

Previously, we demonstrated that elevated expression of *impA1* in *Dictyostelium* cells reverses Li^+^ inhibition of chemotaxis [19]. This is consistent with the uncompetitive inhibition mechanism of Li^+^ on IMPase, which unlike other modes of inhibition is only reversed by increased enzyme concentration. This mechanism alone offers an explanation for how PO inhibition could confer Li^+^ resistance, however our observations on gene expression suggest a more extensive effect of PO on expression of multiple inositol synthetic genes. This could combine to enhance Li^+^ resistance. We investigated cells with elevated expression of both *impA1* and *ino1* and found that they appeared to be Li^+^ resistant. Quantification showed that in terms of cell speed, *impA1:ino1* over-expressing cells were Li^+^ resistant in comparison to wild type cells. However, we also observed that these cells exhibited higher cell speed compared to wild type cells in the Na^+^ control treatment (Figure 5 A,B). In contrast, Directionality was reduced in both Na^+^ control and Li^+^ treated cells, effectively making the cells more sensitive than wild type with regard to this parameter. These observations demonstrate that whilst mis-regulation of multiple genes may enhance overall Li^+^ resistance, there is likely to be more complexity to the regulation, either involving more genes than *ino1* and *impA1* or perhaps a finer balance in levels of expression.

### PO and Mipp1 modulate gene expression in HEK293 cells

**Figure 6:**
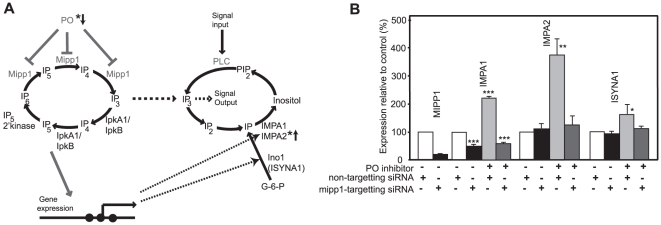
PO regulates expression of human IMPA1, IMPA2 and ISYNA1 genes. **A**, A diagram to illustrate the gene regulatory network through which PO activity regulates expression of the IMPA1, IMPA2 and ISYNA1 genes. The gene regulatory network is separate from ligand-stimulated regulation of IP_3_ signalling via phospholipase C (PLC), but interacts via changes in gene expression or interchange of soluble IP_3_. * marks genes and enzyme activities associated with bipolar mood disorder. **B**, HEK293 cells were treated with either mipp1 specific or non-targetting siRNA for 24 hours with or without 130 µM Z-pro-L-prolinal (PO inhibitor). Gene expression was quantified by qRT-PCR using expression of B2M as a reference. Expression of IMPA1, IMPA2 and ISYNA1 genes was quantified as percentage relative change compared to control (DMSO carrier treated, non-targeted siRNA samples). * p<0.05, ** p<0.02, *** p<0.001.

These results show that PO and Mipp1 control expression of the *Dictyostelium* inositol synthetic genes (Figure 6A). A key question is whether a similar modulatory pathway exists in human cells. We therefore examined the effects of PO inhibition on HEK293 cells. PO inhibition caused a strong elevation of expression of the human IMPase genes, IMPA1 and IMPA2 (Figure 6B). siRNA to knockdown human Mipp1 expression again demonstrated that this increase was dependent on Mipp1. Interestingly, basal IMPA1 expression was reduced by *mipp1* siRNA (Figure 6B), suggesting that in these human cells, IMPA1 expression may be more dependent on Mipp1 function than other co-regulated genes. PO inhibitors also lead to a smaller Mipp1-dependent elevation in expression of the human inositol synthase gene ISYNA1.

## Discussion

These results indicate that PO and Mipp1 are key components of a gene regulatory network that controls expression of the *Dictyostelium* inositol synthetic genes (Figure 6A). We suggest that this may act in *Dictyostelium* during aggregation stage of development to modulate chemotaxis. It has previously been observed that *Dictyostelium* change their chemotactic response during aggregation in order to accommodate changes in cAMP signalling as cells approach multicellularity [28]. Little is known about the mechanisms that mediate these changes, however we observed elevated PO activity during these later stage of aggregation, and note that DpoA appears to act as a suppressor of cell speed during chemotaxis, suggesting that the PO/Mipp1 signal pathway could be involved with these developmental changes.

The rapid elevation of IP_3_ in response to PO inhibition, suggests a series of direct interactions within this signalling pathway, and consistent with this we see no change in *mipp1* mRNA following PO inhibition. Furthermore, we show that incubation with recombinant PO decreases Mipp1 activity in cell extracts. This *in vitro* effect does not proceed via proteolysis of Mipp1, and therefore is likely to involve an unidentified intermediate peptide or protein.

In addition, we show that although I(1,4,5)P_3_ is the Mipp1 product, it is not the modulator of Li^+^ sensitivity. Instead, we show that this occurs via altered expression of the genes that encode the myo-inositol synthetic and metabolic genes. This fits biochemical observations as Li^+^ is an uncompetitive inhibitor of the IMPase and IPP [6], [29] and hence targets the enzyme-substrate complex. Uncompetitive inhibitors can only be overcome by increasing the amount of enzyme, and we have previously shown that increased expression of ImpA1 reduces Li^+^ sensitivity of *Dictyostelium* cells undergoing chemotaxis [19]. We also previously found that in the absence of Li^+^, elevated ImpA1 enhances signalling via PIP_3_, an important mediator of the chemotaxis in *Dictyostelium* and signalling in other cells. We now show that a combined increase in expression of *impA1* and *ino1*genes, as occurs in the *dpoA* null mutant, enhances the degree of Li^+^ resistance with regard to cell speed and also alters the chemotactic response in the absence of Li^+^. A combined up-regulation of gene expression would be expected to have significant effects on PIP_3_ signalling.

The exact nature of the link between Mipp1 and gene expression requires further study, however genetic evidence implicates signalling via changes in the metabolism of the higher order inositol phosphates, I(1,3,4,5,6)P_5_ and I(1,4,5,6,)P_4_ [30]. I(1,3,4,5,6)P_5_ is a good candidate for a signalling and has previously been observed in the control of *ino1* and *pho* gene expression in yeast [25]. In mammals, recent results indicate the I(1,3,4,5,6)P_5_ rapidly accumulates following Wnt3A stimulation [31].

To investigate the effects of PO inhibition in animal cells, we examined PO inhibition and siRNA knock down of Mipp1in HEK293 cells. As found in *Dictyostelium*, expression of *ino1* (ISYNA1), IMPA1 and the IMPase paralogue IMPA2 are increased in a Mipp1 dependent manner following treatment with PO inhibitors. Interestingly, basal IMPA1 expression was reduced by Mipp1 siRNA and no subsequent elevation of expression was seen with PO inhibition. This suggests that in human cells, expression of IMPA1 may have a greater requirement for Mipp1 activity than other genes. We note that although the same dependency is not seen in *Dictyostelium*, we did observe a differential inhibitory effect on *impA1*and *ino1* following Mipp1 over-expression (Figure 4D). It is currently unclear how these differences in expression arise between genes, however we suggest that they reside in subtle differences in their promoter structure.

How might PO mediated signalling be relevant to bipolar mood disorder? First, altered PO activity has been observed in bipolar disorder patients. In manic phase patients prior to treatment, plasma PO activity is elevated, but then reduced to below that of control patients following mood stabilizer treatment [13]. In a follow up study, PO activity was shown to be below that of control groups in euthymic patients [14]. Both sets of measurements were made using plasma, however PO is a cytosolic enzyme and plasma levels of activity are 1,000-fold less than intracellular activity [14]. The relationship between intracellular activity and patients has not yet been explored.

The potential causes of altered PO activity are unknown, but as single nucleotide polymorphism (SNP) analysis has failed to find a genetically significant association of PO with risk of bipolar disorder [32], it may arise from environmental or physiological effects. High doses of the cytokine IFN-α in patients undergoing immunotherapy are reported to decrease serum PO activity and be associated with some symptoms of depression [33], and mood disorders in general have previously been associated with an activation of the inflammatory response. A recent report found bipolar disorder patients have altered expression of a subset of genes that are associated with the pro-inflammatory response, including those involved in cytokine signalling and chemotaxis [34]. Furthermore twin studies showed that these changes arise through a common environmental factor, not a shared genetic component [34]. Altered PO activity may therefore reflect an environmental or physiological component for risk of bipolar disorder.

Second, genetic studies suggest that the presence of a specific set of SNPs within the IMPA2 gene promoter associates with elevated risk for bipolar disorder in some family studies [35], [36], [37]. Some of these SNPs have been shown to elevate promoter activity when coupled to marker genes and may also associate with elevated expression in post-mortem brain samples, although these studies may be confounded by differences in brain pH [37]. Global differences in brain IMPase activity have not been substantiated [38], [39], although differences could be missed by sampling different populations, or if changes only occur in a small subset of cells or brain regions.

Third, we have identified a novel signal pathway, where PO acts to suppress gene expression of the inositol synthetic and metabolic genes. It seems significant that PO suppresses IMPase gene expression, which is a Li^+^ target. In *Dictyostelium*, loss of PO leads to reduced Li^+^ sensitivity. This would seem the opposite case to that seen in patients, where Li^+^ treatment suppresses a clinical state. However, the difference may be that Li^+^ treatment of *Dictyostelium* cells leads to a quantifiable difference in cell behaviour, whereas no behavioural difference has been observed in human non-patient controls. Our *Dictyostelium* experiments can be viewed from an alternative perspective, where our mutant *Dictyostelium* cells possess aberrant signalling and in some cases exhibit altered chemotaxis behaviour. Li^+^ treatment suppresses this aberrant signalling behaviour so that it now falls into the behavioural parameters measured in wild type cells prior to Li^+^ treatment. For example in the case of both the *dpoA* null mutant and *ino1:impa1* over-expressors we observed differences in chemotactic behaviour that were reversed by Li^+^ treatment. Similarly we have previously observed elevated PIP_3_ signalling in wild type cells over-expressing *impa1* in the absence of Li^+^, which is suppressed by Li^+^ treatment [19].

We therefore envisage that treatment of patients may fit a general model, where elevated signalling activity is suppressed by mood stabilizer treatment. We propose that altered transcriptional control of inositol synthetic and metabolic genes, particularly IMPA2, could contribute to elevated risk of bipolar disorder. This risk could be heightened through changes in PO activity. In the simplest scenario, a genetic predisposition for elevated IMPA2 expression could be enhanced though an environmentally mediated decrease in PO activity leading to overactive cell signalling. Long-term Li^+^ treatment would in turn suppress elevated cell signalling to compensate for elevated gene expression. Although this will be difficult to directly demonstrate in patient studies, further genetic analysis and development of model systems will be useful to probe the clinical relevance of PO-mediated signalling.

## Materials and Methods

### Analysis of Cell movement

All *Dictyostelium* mutants and over-expressing strains were generated in the AX2 parental background (Materials and Methods S1) and grown using standard methods [40]. Cells were shaken at 10^6^ cells/ml for 5 hours in KK2 with 6-minute pulses of 100 nM cAMP every 6 minutes. Lithium was added for the final hour of pulsing. Cells were placed in 1 µM gradient of cAMP[41] and images collected every 10 seconds on a DIC inverted microscope. Data was analysed using DIAS 3.4.1 analytical software (Soll Technologies Inc; [42]). Data sets were analysed for statistical significance using a Mann-Whitney U-test, and based on 200–300 cells per experiment.

#### Biochemical analysis

PO activity was measured as described by Williams et al (1999) [11]. For MIPP activity, cell extracts were prepared as described by Van Dijken et al (1995) [43] and incubated with an equal volume of TEE [20 mM triethanolamine pH 6.5, 5.9 mM EGTA, 0.5 mM EDTA] supplemented with 20 mM CaCl_2_, 50 mM LiCl and 200 µM IP_6_. Production of IP_3_ was measured by an isotope dilution assay (Perkin Elmer). For HPLC-MDD analysis, IP were prepared from 10^9^ cells and resuspended in 400 µl 0.1 M NaCl, 15 mM NaF, 0.5 mM EDTA pH 6.0, and quantified by hplc analysis using Tricorn Mini Q 4.6/50 PE columns (GE healthcare) with solutions A: (21 µM YCl_3_) and B (0.8 M HCl, 25 µM YCl_3_ (Merck). Post-column detection used (2.13 M triethanolamine, 500 µM 4-(2′-Pyridylazo)-Resorcinol (PAR) (Merck), pH 9.75) and absorbance [44] at 520 nm [45], [46].

### Analysis of gene expression

Northern blotting was done using standard techniques. For qRT-PCR analysis of *Dictyostelium* samples, total RNA was extracted from growing cells and used as template to generate cDNA using random primers (Roche 1^st^ Strand synthesis kit). qRT-PCR was then performed using a SYBR green mastermix kit (Abgene), with 10 ng cDNA template and PCR primers described in Materials and Methods S1. All samples were run in triplicate, with at least three biological replicates. Expression differences were calculated using the 2^−ΔΔcT^ method [47] normalised to *rnlA* in *Dictyostelium* and B2M in HEK 293 cells.

## Supporting Information

Materials and Methods S1 Construction of mutant strains and plasmids; qRT-PCR primers.
(0.05 MB DOC)

Table S1 Summary of the genes identified and manipulated in this study. mRNA copy number was determined by quantitative RT-PCR, by using a calibration curve generated from plasmid clones of each gene, assuming that 1% of total RNA is mRNA. The activity of genes marked * is predicted by homology with characterised enzymes from other species. [Williams RS, Eames M, Ryves WJ, Viggars J, Harwood AJ (1999) EMBO J 18: 2734–2745; Fischbach A, Adelt S, Muller A, Vogel G (2006) Biochem J. 397:509–518.; King JS, Teo R, Ryves J, Reddy JV, Peters O, Harwood, AJ. (2009) Dis Model Mech 2: 306–312.; Loovers HM, Veenstra K, Snippe H, Pesesse X, Erneux C, et al. (2003) J Biol Chem 278: 5652–5658].
(0.08 MB DOC)
