# Supplementary material for: Genetic Control of Lithium Sensitivity and Regulation of Inositol Biosynthetic Genes
Source: PLoS One. 2010 Jun 17;5(6):e11151. doi: 10.1371/journal.pone.0011151 (PMC2887444; doi:10.1371/journal.pone.0011151)
Supplement: Materials and Methods S1 — Construction of mutant strains and plasmids; qRT-PCR primers. (0.05 MB DOC) [file pone.0011151.s001.doc]

**Supplementary Materials and Methods**

**Generation of mutants**

A full-length clone of *mipp1* generated by PCR from genomic DNA using the primers caCAAACCAACTatgatggtaaaaat / ttattggatcaatggagatcttga was cloned into the TOPO blunt II vector (Invitrogen). This was truncated by digestion with BglII and BamHI and the blasticidin selection cassette from pBLPBSR [1] was inserted. For *mipp1*/*dpoA* double mutants, *mipp1* null cells the Bsr gene was removed by transformation with a NLS-cre recombinase plasmid and blasticidin-sensitive clones transformed with the dpoA knockout plasmid [2].

**Over-expression of Mipp1, IpkA1, IpkB, ImpA1 and ino1**

A *mipp1* overexpression plasmid was generated by PCR with the primers GGATCCcaCAAACCAACTatgatggtaaaaat

gcggccgcttattggatcaatggagatcttgaatc,

adding 5’ BamHI and 3’ NotI restriction sites and cloned into pRHI8 (gift from R. H. Insall). Full length genomic clones of IpkA1 and IpkB were generated by PCR with the primers GGATCCATGAATGACCAAAATAAATTTCAAG

CTCGAGTTTTTGTGGTTGTTGAGGTTGTTG

and

GGATCCATGGGTCGAAATAATACATTAC

GTTTTTATACCGATGATTTTCTT

and cloned into TOPO blunt II, excised with BamHI/Xho I and ligated into pTX-FLAG [3]. Plasmids were transformed into *Dictyostelium* and selected on G418. An N-terminal GFP-fusion of IMPase was generated using the full-length cDNA clone FCBP15 obtained from the Japanese *D. discoideum* cDNA project [4]. The full coding region was amplified by PCR using the primers

AGATCTAAAATGGAAAATATTACTCTTGATCAATAC

and

TGTAAAACGACGGCCAGT

and sub-cloned into the BluntII Topo vector (Invitrogen). An N-terminal GFP-fusion of *Ino1* was generated using the cDNA clone SLB678 obtained from the Japanese *D.discoideum* cDNA project 3 using the primer pair

AGATCTATAATGTCAGCACAAATGTTTGAATCATTCAAG

and

CTCGAGCTAAACAAAAATGAATACCAATATTTAAATAAC

and cloned into the BluntII Topo vector (Invitrogen). Both impA1 and ino1 genes were excised and ligated as a *Bgl* II-*Xho* I fragment into pTX-GFP 2. Plasmids were transformed into *Dictyostelium* and selected on G418.

**RT-PCR primers for quantitative realtime PCR (qRT-PCR)**

**A: Dictyostelium**

The following primers were used for q-RT-PCR:

*ino1*

GACACCGTCGTCGTTATGTGGTCCG/ gcaatcatatcatcgacaacatttg;

*impA1* ggtaatgaaccaacatgggtaattg/gctgtacctgagaatcttaatgcttgaac; *ippA*

AGAGGAACAATGTGAAATACCAAC/ TTGAACTTTATCATTGAAATGAGAGGC;

*ippB* gatcaagaggaaccaattaaggtttc/ ccttttgagtaatctaattgttgttt; *Dd5P2* ggttcaaatcagtatggtcaattgggattg/GTGAAAACATCTTGTTCCTTTGTGAGAAGG;

*Dd5P3* ACGATTGGTGTTTCCCAATTATACAAGG/TGCAATTGAAAAGCTGGTGTTGTCGC; *Dd5P4* GTATTATCAATTAGTGATCAATCAACACC;

*mipp1* ACCAATGAAATACAAACCACAATCC/ TCCATCCACTCCCAATGAACC;

*ipkA1* GCAGGTTCAACACCATTCAAAAAATC/ TCCAACACTATCCATTCCTTACCATC; *ipkA2* TGGTAGTTTTTTGAGTGTCAGCCC/ TGATGATGTTGTTGTTGTTGTTGTAGTG; *ipkB* TTACAGTTGGCTACTACTTGTCAGATTCC/ atggattatcaaggtcacctaactcatca;

*dpoA* GTGGTCAATCTACAAATGCTCAATCG/GCATTAAAGGTTCTCTTAACGTGG;

*rnlA* TCCAAGAGGAAGAGGAGAACTGC/TGGGGAGGTCGTTACACCATTC.

**B: HEK293**

IMPA1 CAGTTGTGTGGAAGGCAAGA/TGTTCTGGAAGAGCCCAACT

IMPA2 TCAGGGCCAAAACTCAAATC/ GAACGATCCGCTTTATCAGC

ISYNA1 ACCAAAGTCAAGTCCGTGCT/TTGCTCTTGGACACCTCCTT

B2M TGCTGTCTCCATGTTTGATGTATC/ TCTCTGCTCCCCACCTCTAAG

**References**

1. J. Faix, L. Kreppel, G. Shaulsky et al., (2004) A rapid and efficient method to generate multiple gene disruptions in *Dictyostelium discoideum* using a single selectable marker and the Cre-loxP system. Nucleic Acids Res 32: e143.

2. Williams RS, Eames M, Ryves WJ, Viggars J, Harwood AJ (1999) Loss of a prolyl oligopeptidase confers resistance to lithium by elevation of inositol (1,4,5) trisphosphate. EMBO J. 18: 2734-2745

3. S. Levi, M. Polyakov, and T. T. Egelhoff. (2000) Green fluorescent protein and epitope tag fusion vectors for *Dictyostelium discoideum*. Plasmid 44 : 231.

4. T. Morio, H. Urushihara, T. Saito et al. (1998) The *Dictyostelium* developmental cDNA project: generation and analysis of expressed sequence tags from the first-finger stage of development. DNA Res 5: 335.
