# Supplementary material for: Genetic Control of Lithium Sensitivity and Regulation of Inositol Biosynthetic Genes
Source: PLoS One. 2010 Jun 17;5(6):e11151. doi: 10.1371/journal.pone.0011151 (PMC2887444; doi:10.1371/journal.pone.0011151)
Supplement: Table S1 — Summary of the genes identified and manipulated in this study. mRNA copy number was determined by quantitative RT-PCR, by using a calibration curve generated from plasmid clones of each gene, assuming that 1% of total RNA is mRNA. The activity of genes marked * is predicted by homology with characterised enzymes from other species. [Williams RS, Eames M, Ryves WJ, Viggars J, Harwood AJ (1999) EMBO J 18: 2734–2745; Fischbach A, Adelt S, Muller A, Vogel G (2006) Biochem J. 397:509–518.; King JS, Teo R, Ryves J, Reddy JV, Peters O, Harwood, AJ. (2009) Dis Model Mech 2: 306–312.; Loovers HM, Veenstra K, Snippe H, Pesesse X, Erneux C, et al. (2003) J Biol Chem 278: 5652–5658]. (0.08 MB DOC) [file pone.0011151.s002.doc]

| **Gene** | **Enzyme** | **Predicted activity** | **E.C. number** | **Accession no.** | **Reference** | **mRNA copy number /cell** |
| --- | --- | --- | --- | --- | --- | --- |
| *dpoA* | Prolyl oligopeptidase (PO) | Peptidase | E.C. 3.4.21.26 | DDB0185041 | Williams *et al.* 1999 | 5 |
| *ino1* | Inositol synthase | InsP_1_ synthase | E.C. 5.5.1.4 | DDB0231710 | Fischbach *et al.* 2006 | 100 |
| *impA1* | Inositol monophosphatase (IMPase) | InsP_1_ phosphatase | E.C. 3.1.3.25 | DDB0204100 | King *et a*l  2009 | 50 |
| *ippA* | Inositol polyphosphate phosphatase (IPPaseA) | InsP_2_ phosphatase* | E.C. 3.1.3.57 | DDB0167248 | This work | 10 |
| *ippB* | IPPaseB | InsP_2_ phosphatase* | E.C. 3.1.3.57 | DDB0189923 | This work | 10 |
| *Dd5P3* | Inositol 5’phosphatase | Inositol polyphosphate 5’-phosphatase | E.C. 3.1.3.56 | DDB0185010 | Loovers *et al.* 2003 | 5 |
| *mipp1* | Multiple inositol  polyphosphate phosphatase (Mipp1) | InsP_6_ 2’/3’/6’ phosphatase | E.C. 3.1.3.62 | DDB0186447 | This work | 15 |
| *ipkA1* | Inositol polyphosphate  multikinase (IpkA1) | Ins(1,4,5)P_3_-3’/6’ kinase* | E.C. 2.7.1.151 | DDB0203614 | This work | 10 |
| *ipkB* | IpkB | Ins(1,3,4)P_3_-5’/6’-kinase / Ins(3,4,5,6)P_4_-1’ kinase* | E.C. 2.7.1.134 | DDB0190521 | This work | 20 |
| *rnlA* | Mitochondrial large  subunit rRNA | n/a | n/a | DDB0237480 |  | 670 |
|  |  |  |  |  |  |  |

**Table S1. Summary of the genes identified and manipulated in this study.** mRNA copy number was determined by quantitative RT-PCR, by using a calibration curve generated from plasmid clones of each gene, assuming that 1% of total RNA is mRNA. The activity of genes marked * is predicted by homology with characterised enzymes from other species.

[Williams RS, Eames M, Ryves WJ, Viggars J, Harwood AJ (1999) EMBO J 18: 2734-2745; Fischbach A, Adelt S, Muller A, Vogel G (2006) Biochem J. 397:509–518.; King JS, Teo R, Ryves J, Reddy JV, Peters O, Harwood, AJ. (2009) Dis Model Mech 2: 306-312.; Loovers HM, Veenstra K, Snippe H, Pesesse X, Erneux C, et al. (2003) J Biol Chem 278: 5652-5658]
